# Clinical Grade Purification and Expansion of NK Cell Products for an Optimized Manufacturing Protocol

**DOI:** 10.3389/fonc.2013.00118

**Published:** 2013-05-17

**Authors:** Ulrike Koehl, Claudia Brehm, Sabine Huenecke, Stefanie-Yvonne Zimmermann, Stephan Kloess, Melanie Bremm, Evelyn Ullrich, Jan Soerensen, Andrea Quaiser, Stephanie Erben, Claudia Wunram, Tanja Gardlowski, Eileen Auth, Torsten Tonn, Christian Seidl, Sandrine Meyer-Monard, Martin Stern, Jakob Passweg, Thomas Klingebiel, Peter Bader, Dirk Schwabe, Ruth Esser

**Affiliations:** ^1^Institute of Cellular Therapeutics, Integrated Research and Treatment Center Transplantation, Hannover Medical SchoolHannover, Germany; ^2^Pediatric Hematology and Oncology, J. W. Goethe-UniversityFrankfurt, Germany; ^3^Center for Cell and Gene Therapy, J. W. Goethe-UniversityFrankfurt, Germany; ^4^Red Cross Blood Donor CenterFrankfurt and Dresden, Germany; ^5^Stem Cell Transplant Team, Basel University HospitalBasel, Switzerland

**Keywords:** NK cell purification, NK cell expansion, cytotoxicity, IL-2 activation, T-cell removal

## Abstract

Allogeneic natural killer (NK) cells are used for adoptive immunotherapy after stem cell transplantation. In order to overcome technical limitations in NK cell purification and activation, the following study investigates the impact of different variables on NK cell recovery, cytotoxicity, and T-cell depletion during good manufacturing practice (GMP)-grade NK cell selection. Forty NK cell products were derived from 54 unstimulated donor leukaphereses using immunomagnetic CD3 T-cell depletion, followed by a CD56 cell enrichment step. For T-cell depletion, either the depletion 2.1 program in single or double procedure (D2.1_1depl_, *n* = 18; D2.1_2depl_, *n* = 13) or the faster depletion 3.1 (D3.1, *n* = 9) was used on the CliniMACS instrument. Seventeen purified NK cell products were activated *in vitro* by IL-2 for 12 days. The whole process resulted in a median number of 7.59 × 10^8^ CD56^+^CD3^−^ cells with both purity and viability of 94%, respectively. The T-cell depletion was significantly better using D2.1_1depl/2depl_ compared to D3.1 (log 4.6/log 4.9 vs. log 3.7; *p* < 0.01) and double procedure in two stages led always to residual T cells below 0.1%. In contrast D3.1 was superior to D2.1_1depl/2depl_ with regard to recovery of CD56^+^CD3^−^ NK cells (68% vs. 41%/38%). Concomitant monocytes and especially IL-2 activation led to increased NK cell activity against malignant target cells compared to unstimulated NK cells, which correlated with both up-regulation of natural cytotoxicity receptors and intracellular signaling. Overall, wide variations in the NK cell expansion rate and the distribution of NK cell subpopulations were found. In conclusion, our results indicate that GMP-grade purification of NK cells might be improved by a sequential processing of T-cell depletion program D2.1 and D3.1. In addition NK cell expansion protocols need to be further optimized.

## Introduction

Natural killer (NK) cells play an important role in the immune response against leukemia or tumor cells after stem cell transplantation. They represent a promising therapeutic option for patients with various types of malignant disease (Passweg et al., 2006; Rubnitz et al., 2010). NK cells are able to exert a graft-vs.-leukemia/tumor (GvL/T) effect without concomitant severe graft vs. host disease (GvHD) (Ruggeri et al., 2002). Phenotypically, they express CD56, an isoform of the neural cell adhesion molecule, on their surface simultaneously lacking the CD3 antigen. They can be further divided into a CD56^dim^CD16^+^ population of about 90% that has cytotoxic activity, and a CD56^bright^CD16^dim/−^ subpopulation with immunoregulatory properties. They proliferate in response to IL-2 and produce large quantities of cytokines, among these IFN-γ, TNF-α, TNF-β, IL-10, and IL-13. A set of surface receptors on each cell can either induce or inhibit the cytotoxic response. Compared to resting NK cells, cytotoxicity of IL-2 activated NK cells is enhanced by up-regulation of the natural cytotoxicity receptors (NCRs) NKp30, NKp44, NKp46, and the NK group 2D (NKG2D) receptor (Moretta et al., 2001; Farag et al., 2002; Lanier, 2005; Ljunggren and Malmberg, 2007).

Adoptive immunotherapy with unstimulated or IL-2 activated donor NK cell infusions (NK-DLI) is currently used in patients with high risk of relapse after haploidentical stem cell transplantation (haploSCT) (Rubnitz et al., 2010; Brehm et al., 2011; Stern et al., 2013). Preliminary results are encouraging. Yet, crucial issues remain unanswered, among these, both, clinical questions (i.e., suitable target diseases and timing of NK-DLI) and questions regarding the processing of the original cell product (i.e., optimal T-cell depletion and NK cell enrichment). Although first protocols for clinical grade NK cell enrichment and culture observing good manufacturing practice (GMP) have been established, so far, no standard procedure has been defined.

Here, we evaluate variables that influence T cell depletion and overall purity, expansion, and cytotoxic activity of clinical grade NK cell products.

## Materials and Methods

### NK cell study

Both, children and adults with high-risk malignancies received unstimulated NK-DLIs at days +3, +40, and +100 or IL-2 stimulated NK-DLI at days +40 and +100 following haploSCT as described previously (Koehl et al., 2005; Brehm et al., 2011; Stern et al., 2013). For this purpose, we enriched 54 apheresis products from 33 healthy donors, aiming at a cell dose of greater than or equal to 1 × 10^7^ CD56^+^CD3^−^ NK cells/kg with less than 1 × 10^5^ CD3^+^ T cells/kg recipient’s body weight (BW) at each timepoint. Informed consent was obtained from the donors after approval of the local ethics committee for both a small NK-DLI feasibility study and a subsequent bi-center phase I/II trial registered at clinicaltrials.gov (NCT01386619). Apheresis products were collected from 22 male and 18 female donors. Median age and BW of the donors were 39 years (range: 27–53) and 75 kg (range: 62–123), respectively.

The apheresis products underwent a single or double CD3 depletion step detailed below followed by a CD56 enrichment step. Seventeen NK cell products were activated and expanded with 1000 U/ml rhIL-2. GMP was observed throughout.

### Clinical grade NK cell purification

**Figure 1:**
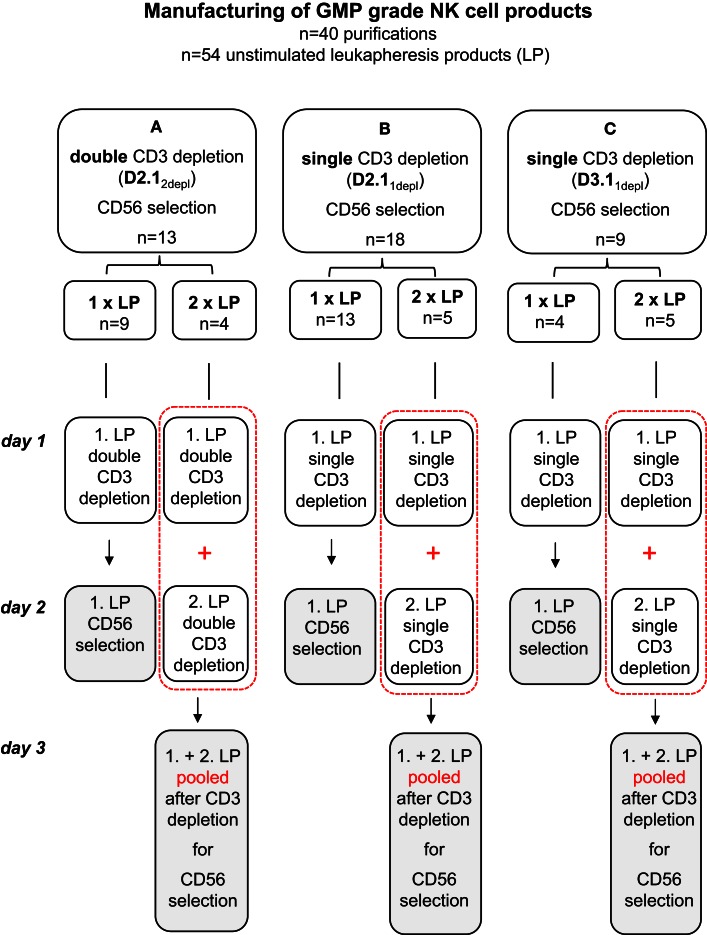
**Manufacturing of GMP-grade NK cell products**. To optimize manufacturing of NK cell products three different methods were used for purification: **(A)** two T-cell depletion steps using the CliniMACS depletion program 2.1 (D2.1_2depl_), **(B)** one T-cell depletion step with the same program (D2.1_1depl_), and **(C)** one T-cell depletion step using the CliniMACS depletion program 3.1 (D3.1_1depl_). Unstimulated leukapheresis products were collected and CD3^+^ depletion followed immediately. If two leukapheresis products were available on day 1 and day 2, both CD3 depleted products were pooled prior to the CD56 enrichment. In A, an additional T-cell depletion step was ensued. Finally in all cases a CD56^+^ positive selection was done with the CD3 depleted products using the CliniMACS enrichment program E1.1.

Fifty-four steady-state leukaphereses were carried out using a Cobe Spectra cell separator (Gambro BCT, Munich, Germany). In 26 cases, we processed a single leukapheresis product, while in 14 cases, 2 leukapheresis products each were pooled. To optimize manufacturing of NK cell products three different methods were used for purification as shown in Figure 1; (A) two T-cell depletion steps using the CliniMACS depletion program 2.1 (D2.1_2depl_), (B) one T-cell depletion step with the same program (D2.1_1depl_), and (C) one T-cell depletion step using the CliniMACS depletion program 3.1 (D3.1_1depl_). Clinical grade enrichment of NK cells was accomplished by CD3^+^ depletion followed by CD56^+^ selection. In case that we processed a single leukapheresis product, the total enrichment procedure lasted two working days. In the cases that two leukapheresis products were processed, each underwent CD3^+^ depletion singly. The first was depleted immediately or in exceptional cases the next morning after apheresis, the second immediately on the day after collection. Then, the products were pooled. In 13 cases, an additional T-cell depletion step ensued. Afterward, CD56^+^ positive selection was done.

In detail: for the actual immunomagnetic procedure, an unstimulated leukapheresis product was washed twice to remove platelets with CliniMACS buffer (Miltenyi Biotech, Bergisch Gladbach, Germany) supplemented with 0.4% human serum albumin (Red Cross Blood Donor Service, Baden-Württemberg-Hessen, Germany). Then, 5 ml of Intraglobin (Biotest, Dreieich, Germany) was added and incubated for 5 min to reduce non-specific antibody binding. Subsequently, we added the CliniMACS CD3 Reagent (Miltenyi Biotec) and incubated the cells for 30 min, using one vial of reagent for either total nucleated cell (TNC) counts of up to 4 × 10^10^ and CD3^+^ cell counts of up to 1.5 × 10^10^. An additional vial was employed for up to twice the numbers. After two washing steps, CD3^+^ cells were immunomagnetically depleted on the CliniMACS instrument using either the program “DEPLETION 2.1” and a LS tubing set (Miltenyi Biotech) or the program “DEPLETION 3.1” and a DTS tubing set (Miltenyi Biotech). A second depletion run of the NK cell containing negative fraction without additional labeling step was employed to remove any residual T cells in 13 of the 31 cases that we used the program “DEPLETION 2.1,” while in 18 of 31 instances, only one T-cell depletion run was done. In the cases that we used “DEPLETION 3.1,” no additional depletion run was performed. Finally, the T-cell depleted cell fraction was concentrated and labeled with clinical grade CD56 MicroBeads^®^ (MiltenyiBiotech) for 30 min (one vial for TNC numbers of up to 4 × 10^10^ and CD56^+^ cell numbers of up to 1 × 10^10^) and washed. Then, the CD56^+^CD3^−^ NK cells were enriched using the program “ENRICHMENT 1.1.” All steps were performed in a closed system according to GMP.

### Expansion and activation of NK cells

The purified CD56^+^CD3^−^ NK cells were suspended at 1 × 10^6^ cells/ml in X-VIVO 10 media (Lonza, Basel, Switzerland) supplemented with 5% heat-inactivated human fresh frozen plasma (FFP) and 1000 U/ml rhIL-2 (Proleukin^®^ Novartis Pharma Nürnberg, Germany) for up to 12 days in GMP-grade VueLife^®^ culture bags (Cellgenix, Freiburg, Germany). In the context of feasibility assessment, the cells from the first three donors, only, were cultured in 175 cm^3^ culture flasks (Nunc, Wiesbaden, Germany). In each case, fresh media were added every 3 days. To optimize NK cell expansion, we carried out a series of small scale experiments comparing the use of 1000 U/ml rhIL-2 with combined IL-2 (100 U/ml) and IL-15 (10 ng/ml) stimulation. For cryopreservation, NK cells were concentrated and resuspended in X-VIVO 10 media diluted 1:2 with 20% dimethyl sulfoxide (DMSO).

### Flow cytometric quality control analysis

**Figure 2:**
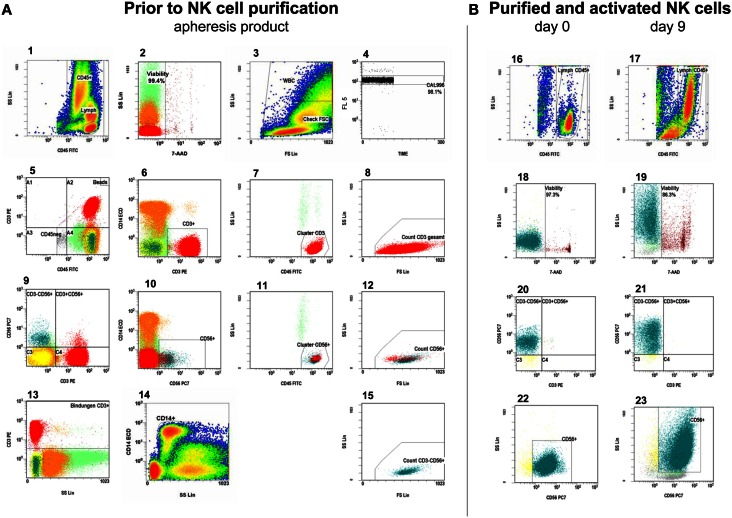
**Measurement of absolute viable CD56^+^CD3^−^ NK cells and CD3^+^ T cells**. **(A)**. Gating strategy in the leukapheresis product: from left to right, starting in the first row of plots, the gating strategy follows the ISHAGE protocol for stem cell enumeration (Keeney et al., 1998), modified for the peculiarities of NK- and T-cell measurement: plot 1: the region is set to include all CD45^+^ events (leukocytes) and is gated on viable cells (plot 2). Therefore, only 7-AAD negative (vital) events are shown in plot 1. Region: CD45^high^, SC^low^ events: lymphocytes. Plot 2: gated on CD45^+^ events (region, plot 1), the region is set to discriminate between viable and unstained and non-viable cells, which are 7-AAD-positive. Plot 3: all viable CD45^+^ events are shown to check the lower limit of forward scatter (check FSC). The amorphous region is created to exclude unspecifically stained debris by low forward scatter signal. Thus, region WBC includes all viable leukocytes. Plot 4: it displays the fluorescent signal of the events vs. time and is gated on beads (see plot 5, beads gate, top right). Region CAL is set to define the signal of Flow-Count^TM^ fluorospheres and to monitor the occurrence of fluorospheres doublets, which is less than 5% in this plot. CAL is the calibrator region to automatically calculate the concentrations of the events in a given gate. Steady sample flow is monitored here, too. Plot 5 and plot 9: these dot plots show all events and are used as a visual guide for antigen expression of CD3 and CD56. The respective CD45 negative region is used to exclude CD45 negative events in order to reduce the data acquisition. Quadrant 2 can be useful to review the lower limits of CD45 and CD56 or CD3 expression, and – if necessary – to correct the position of the regions in plot 1, 6, and 10. Plot 6 and plot 10: the region includes all viable CD3^+^ or CD56^+^ leukocytes and is gated on the CD45^+^ region in plot 1. Plot 7 and plot 11: logical AND linkage (intersection) of regions CD45^+^ (plot 1), CD3^+^ or CD56^+^ (plots 6, 10) and viability (plot 2). The cluster region is set to include CD45^high^SC^low^ CD45^+^, 7-AAD^−^ CD3^+^ or CD56^+^ events, respectively and to exclude granulocytes. Plot 8, plot 12, and plot 15: intersection of the regions from the regions in plots 1, 2, 6 or 10, and 7 or 11, respectively. The respective region is linked with region check FSC (plot 3) so that changes in the position of region check FSC will automatically be adopted by region in the plots of 8, 12, and 15. Region FSC is used to set the lowest limit for FSC. The thus accepted viable CD3^+^ T cells (plot 8), viable CD56^+^ NK and NK like T cells (plot 12), and the viable CD56^+^CD3^−^ NK cells (plot 15) are counted in regions of the plots 8, 12, and 15, respectively. Plot 13: control gate for showing all, specific and unspecific CD3 antibody binding for the calculation of sufficient CliniMACS CD3 reagent for the CD3 depletion of the NK cells. Plot 14: this is used for both enumeration of CD14^+^ monocytes and as dump channel to exclude monocytes from all analyses, which is of major importance to evaluate residual T cells after NK cell purification. **(B)**. Respective analyses in the purified NK cell products as well as cultured, expanded NK cells: plot 16 and plot 17: the region is set to include all CD45^+^ events in accordance to plot 1. Plot 18 and plot 19: discrimination between viable and dead cells in accordance to plot 2. Plot 20 and plot 21: overview of CD56^+^CD3^−^ NK cells, residual CD3^+^CD56^−^ T cells, and CD56^+^CD3^+^ NK like T cells in accordance to plot 9. Plot 22 and plot 23: the region includes all viable CD56^+^CD3^−^ NK cells gated on the CD45^+^ region (plot 16, 17).

Samples were drawn after leukapheresis and after each depletion and selection as well as every third day during stimulation to monitor cell content and viability. Phenotyping and evaluation of cytotoxicity was performed by flow cytometry. We evaluated the samples for NK cell purity, cell viability, CD56^dim^CD16^+^ and CD56^bright^CD16^dim/−^ NK cell subpopulations, residual T cells, monocytes, dendritic cells (DCs) including the subtypes myeloid DCs (mDCs) and plasmacytoid DCs (pDCs), and NK cell cytotoxic activity. Absolute cell counts were measured by a single-platform approach using Flow-Count™ fluorospheres (Beckman Coulter, Marseille, France). NK and T cells were gated as previously published according to an adapted version of the ISHAGE single-platform stem cell enumeration method using low scatter, high expression of CD3 and CD45 antigens, CD56 expression, and 7-amino-actinomycin D (7-AAD) for assessment of viability (Koehl et al., 2008). An automated lyse/no-wash procedure was used with a fixation step on a TQ-Prep™ Workstation (Beckman Coulter, Krefeld, Germany). CD45-FITC/CD3-PE/CD14-ECD/7-AAD/CD56-PC7 (Figure 2A) and CD45-FITC/CD3-PE/CD16-ECD/7-AAD/CD56-PC7 stained samples were prepared in triplicate or duplicate and CD45-FITC/IgG1-PE/CD14-ECD/7-AAD/CD56-PC7 staining served as control for residual T-cell detection. In addition, a number of samples were labeled with appropriate combinations of fluorochrome-conjugated monoclonal antibodies (MAb) to monitor DCs, the surface expression of NCRs and CD69, phosphorylation of the signal transducer and activator of transcription 3 (STAT3) and the protein kinase AKT. Surface expression of the NCRs NKp30, NKp44, NKp46 on NK cells was quantified in Antibody Binding Capacity (ABC) units by using the *Quantum Simply Cellular* kit (Bangs Laboratories, Indianapolis, IN, USA) as described previously (Huenecke et al., 2010). CD45^+^CD85k^+^HLA-DR^+^CD14^−^CD16^−^CD33^+^ and CD45^+^CD85k^+^HLA-DR^+^CD14^−^CD16^−^CD123^+^ were used to quantify mDCs and pDCs, respectively (Heinze et al., 2013). To analyze the samples, we used cytometers with four and five color detection (Epics™ XL™ and FC500, Beckman Coulter, Krefeld, Germany). Data were analyszed with the aid of CXP v2.2 software (Beckman Coulter, Krefeld, Germany.) MAb conjugated with fluorescein isothiocyanate (FITC), phycoerythrin (PE), PE-Texas Red tandem (ECD), PE-cyanine-5 (PC-5), or PE-cyanine-7 (PC-7) were used against the following antigens (clones): CD3 (UCHT1) and (SK7)#, CD14 (RMO52), CD14^+^CD16 (RMO522^+^3G8), CD16 (3G8), CD33 (D3HL60.251), CD45 (B3821F4A and J.33), CD56 (N901) and (NCAM16.2)#, CD69 (TP1.55.3), CD85k/ILT-3 (ZM3.8), CD123 (107D2), CD335/NKp46 (BAB281), CD336/NKp44 (Z231), CD337/NKp30 (Z25), CD314/NKG2D (ON72), p-STAT-3 (Tyr705), p-AKT (Ser473) (Beckman Coulter, Marseille, France exept # BD Biosciences, Heidelberg, Germany).

### Cytotoxicity assay

The cytotoxic activity of the purified NK cells before and after IL-2 stimulation was tested against the MHC class I-negative cell line K562 and, if available, against the patients’ own leukemic cells using an antibody-based flow cytometric single-platform assay as described previously (Zimmermann et al., 2005; Kloss et al., 2007). NK cells and target cells were co-cultured for 4 h at 1:1 up to 10:1 effector:target ratios. Absolute cell counts were calculated using Flow-Count™ fluorospheres as internal standard. Cytotoxicity was defined as the loss of viable target cells in relation to the mono-cultured control.

### KIR and HLA genotyping

We used a PCR method to detect the presence or absence of 19 killer-cell immunoglobulin-like receptor (KIR) genes (2DL1-5B, 3DL1-3, 2DS1-5, 3DS1, 2DP1, 3DP1) in the peripheral blood cells of both, NK cell donors and patients with sequence-specific primers as described previously (Becker et al., 2003). Moreover, HLA typing was done with sequence-specific probes and sequence-based typing (SBT) analysis. We then evaluated the donor-recipient pairs for the presence of KIR – HLA-ligand mismatches according to the “missing KIR ligand” model.

### Statistical analyses

Statistical analyses were performed using GraphPad Prism 5.03 (GraphPad Software, San Diego, CA, USA). Differences between groups were examined for statistical significance using the Kruskal–Wallis test with Dunn’s multiple comparisons or an unpaired *t*-test. Differences were considered significant when *p* < 0.05, *p* < 0.01, or *p* < 0.001, indicated as *, **, and ***, respectively.

## Results

### Impact of cell processing on NK cell yield, purity, and T-cell removal

A total of 40 apheresis products (26 single, 14 pooled products) were immunomagnetically T-cell depleted, either by the time-consuming, but effective DEPLETION 2.1 program with (A) two consecutive T-cell depletion procedures (D2.1_2depl_, *n* = 13), or (B) by DEPLETION 2.1 program using one T-cell depletion run, only (D2.1_1depl_, *n* = 18), or (C) by using the fast DEPLETION 3.1 program (D3.1, *n* = 9) as indicated in Table 1 and Figure 1.

**Table 1:** **Percentage and absolute count of NK and T cells using different GMP-grade enrichment procedures for NK cell manufacturing**.

	(A) Depletion 2.1 (D2.1_2depl_) 2 CD3 depletion steps (*n* = 13)	(B) Depletion 2.1 (D2.1_1depl_) 1 CD3 depletion step (*n* = 18)	(C) Depletion 3.1 (D3.1_1depl_) 1 CD3 depletion step (*n* = 9)
	Median (range)	Median (range)	Median (range)
**LEUKAPHERESIS PRODUCT**			
WBC (×10^6^)	18907 (4762–74162)	19338 (7608–76680)	21027 (13719–47334)
CD56^+^CD3^−^ NK cells (%)	8.5 (5.4–13.7)	7.5 (2.8–14.9)	7.2 (4.9–15.5)
CD56^+^CD3^−^ NK cells (×10^6^)	1526 (673–2975)	1701 (612–4790)	1628 (693–6156)
CD3^+^ T cells (%)	51 (40–67.7)	56 (43.3–68)	55.7 (45.9–63.7)
CD3^+^ T cells (×10^6^)	10331 (2392–28498)	10056 (3827–43790)	10936 (8594–23557)
**AFTER CD3 DEPLETION**			
WBC (×10^6^)	3822 (982–8560)	5970 (2012–19749)	8356 (4560–20261)
CD5 6 ^+^CD3^−^ NK cells (%)	25 (14–47)	18.9 (6.5–27.1)	17 (10.7–27.8)
CD56^+^CD3^−^ NK cells (×10^6^)	813 (400–2181)	907 (340–3148)	1091 (607–5613)
CD3 ^+^ T cells (%)	0.01 (<0.001–0.08)	0.04 (<0.001–0.29)	0.11 (0.05–0.4)
CD3^+^ T cells (×10^6^)	0.30 (<0.02–2.61)	1.21 (<0.02–17.12)	7.89 (3.83–16.93)
**AFTER CD56 ENRICHMENT**			
WBC (×10^6^)	0.75 (0.25–1.9)	0.83 (0.3–2.9)	0.99 (0.43–4.5)
CD56 ^+^CD3^−^ NK cells (%)	94.9 (82.5–98.3)	92.2 (70–98.2)	93.4 (91.7–97.3)
CD56^+^CD3^−^ NK cells (×10^6^)	712 (234–1880)	745 (284–2703)	942 (426–4254)
CD3^+^ T cells (%)	0.01 (<0.001–0.1)	0.04 (<0.001–0.34)	0.24 (0.07–0.5)
CD3^+^ T cells (×10^6^)	0.12 (<d. l. –1.50)	0.31 (<d. l. −6.99)	3.72 (0.72–5.52)
CD56 recovery (%)	38 (23–69)	41 (14–76)	68 (40–85)
T-cell depletion (log)	4.9 (3.9–5.9)	4.6 (3.2–5.7)	3.7 (3.2–4.1)

*Individual steady-state leukapheresis products: 9 in (A), 13 in (B), and 4 in (C) and pooled products from two leukaphereses: 4 in (A), 5 in (B), and 5 in (C); d. l., detection limit*.

The mean absolute leukocyte numbers and the percentages of T and NK cells did not differ between the three groups. Both, starting numbers of absolute white blood cells (WBC) and percentage of NK cells did not differ between the three groups with median numbers of 1.9 × 10^10^, 1.9 × 10^10^, and 2.1 × 10^10^ for WBCs and 8.5, 7.5, 7.2% for CD56^+^CD3^−^ cells. Absolute numbers of NK cells ranged from 0.67 to 2.98 × 10^9^, 0.61 to 4.79 × 10^9^, and 0.69 to 6.16 × 10^9^, respectively. More than half of the cells were CD3^+^ T cells (median 51, 56, 55.7%).

**Figure 3:**
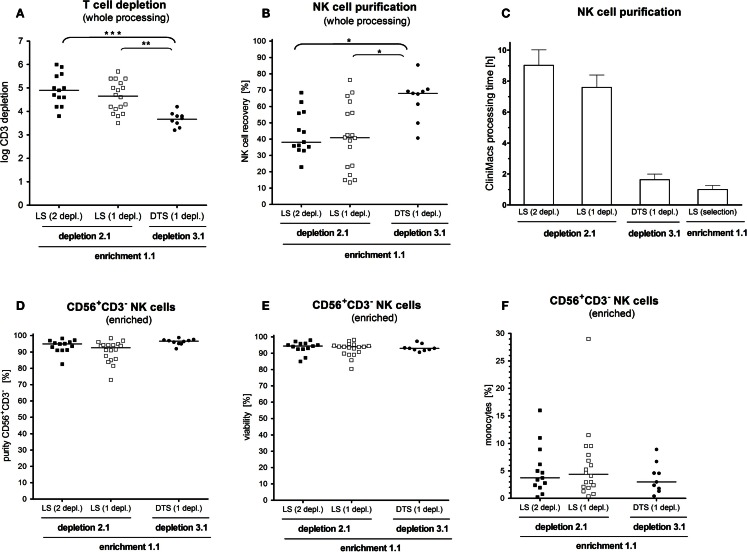
**Impact of cell processing on NK cell yield, purity, and T-cell depletion**. NK cells were enriched by either the depletion 2.1 program in a double T-cell depletion round using an LS purification set (depletion 2.1, 2 depl. *n* = 13), the same program using only one T-cell depletion round (depletion 2.1, 1 depl. *n* = 18) or by using the fast depletion 3.1 program with a DTS purification set (depletion 3.1, 1 depl. *n* = 9). **(A)** T-cell depletion was significant lower with the depletion 3.1 program compared to the depletion 2.1 for one or two rounds of T-cell removal. **(B)** With the depletion 3.1 program a significant higher NK cell recovery was reached compared to the depletion 2.1. **(C)** The NK cell manufacturing on the CLINIMACS device was three to four times higher using the depletion 2.1 program compared to the depletion 3.1. **(D,E)**. Neither purity and recovery did not differ among the three used purification techniques. **(F)**. After the final CD56 enrichment step a large range of concomitant CD14^+^ monocytes (0.3–29.1%) in the respective CD56^+^CD3^−^ NK cell product was observed, which did not differ between the used purification techniques.

After the whole enrichment process, use of D2.1 resulted in significantly better T-cell depletion than D3.1 Two sequential runs of D2.1_2depl_ or one single run of D2.1_1depl_ led to a median T-cell depletion of log 4.9 (range: 3.9–5.9) or log 4.6 (range: 3.2–5.7), respectively, compared to log 3.7 (range: 3.2–4.1) using D3.1 (*p* < 0.001; *p* < 0.01; Figure 3A). The double depletion procedure yielded residual T-cell percentages of less than 0.1% (median 0.01%) as shown in Table 1. The additional CliniMACS depletion run took about 1 h, only, because the cell suspension at that point always contained less than 0.5% CD3^+^ T cells.

In contrast to the effective T-cell removal, D3.1 was superior to both, using single or double D2.1 with respect to recovery of CD56^+^CD3^−^ NK cells (median NK cell recovery 68 vs. 38% and 41%; *p* < 0.05; Figure 3B). All in all, the pure processing time on the CliniMACS device was three to four times longer for D2.1 compared to D3.1 (Figure 3C).

Overall, NK cell enrichment led to a final median number of 7.59 × 10^8^ CD56^+^CD3^−^ cells with both median purity of 94% and viability of 94%. Purity and viability did not differ among the three enrichment protocols (Figures 3D,E). The overall purity ranged from 70 to 98.2% CD56^+^CD3^−^ NK cells. The major population of contaminating cells in the purified NK cell products were CD14^+^ monocytes ranging from 0.2 to 29.6% (Figure 3F). Other than that, we found myeloid and pDCs ranging from 0.1 to 3.8% (data not shown).

### Expansion, activation, and unfreezing of donor derived NK cells

**Figure 4:**
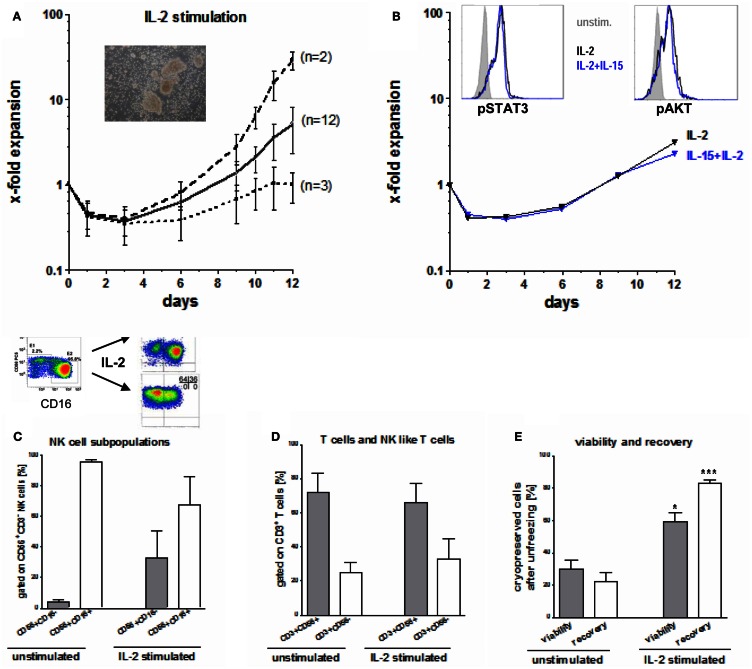
**Expansion, activation, and unfreezing of CD56^+^CD3^−^ NK cells**. **(A)** During the first 4–5 days after IL-2 stimulation, vital NK cell count decreased by 30–65% and started to recover thereafter. Median expansion rate was four times for the allogeneic NK cells of 12 donors, while NK cells of two donors showed a high expansion rate (median 30-fold) and NK cells of three donors reached the starting NK cell count only at the end of the expansion rate. **(B)** Expansion rate during stimulation with 10 ng/ml IL-15 and 100 U/ml IL-2 did not differ from those after stimulation with 1000 U/ml IL-2 only. Intracellular signaling (p-STAT 3 and p-AKT) was increased in stimulated compared to unstimulated NK cells but did not differ between IL-2 or IL-2/IL-15 activation. **(C)** Although the major cytotoxic CD56^dim^CD16^pos^ and the minor immunoregulatory CD56^bright^CD16^neg^ NK subpopulation showed a homogeneous distribution after NK cell enrichment, this changed during IL-2 stimulation. After activation large differences between CD16^pos^ and CD16^neg^ NK subpopulations were seen among the NK cells of various donors. **(D)** After NK cell purification residual T cells consists of 3/4 of CD3^+^CD56^+^ NK like T cells and of 1/4 CD3^+^CD56^−^ T cells, a ratio which changed only slightly during IL-2 stimulation. **(E)** Both, viability and recovery was higher after unfreezing of Il-2 stimulated compared to unstimulated NK cells and was highly significant for the NK cell recovery.

Seventeen NK cell products were further IL-2 stimulated for 10–12 days. During the first 4–5 days of IL-2 stimulation, vital NK cell counts decreased by 30–65% (Figure 4A). Afterward, NK cell counts started to recover. However, we found a wide variation in the NK cell expansion rate among different donors. While the NK cells of two donors expanded vigorously (median 30-fold), those of another three donors recovered to starting NK cell numbers at the end of the expansion period, only (Figure 4A). NK cells of 12 donors expanded moderately to a median fourfold (range: twofold to eightfold). Of note, NK cells tend to grow in clusters (Figure 4A) and need to be singularized carefully for correct quantification, yielding a source for underestimating the true numbers.

Natural Killer cell expansion and intracellular signaling of p-STAT-3 or p-AKT did not differ using IL-2 alone or in combination with IL-2 and IL-15 (Figure 4B). Phosphorylation of the intracellular signaling molecules STAT3 and AKT was augmented in activated NK cells compared to unstimulated NK cells. Initially, we found relatively uniform distribution of the major cytotoxic CD56^dim^CD16^+^ and the minor immunoregulatory CD56^bright^CD16^dim/−^ NK cell subpopulations that changed during IL-2 stimulation as shown in Figure 4C. However, there was a marked variation of the proportions among donors at the end of expansion showing (i) both CD16^+^ and CD16^−^ NK cells, (ii) mainly CD16^−^ or (iii) CD16^+^ NK subpopulations, only.

The residual CD3^+^ T cells at the beginning of IL-2-stimulation consisted to 72 ± 11% of CD3^+^CD56^+^ NK-like T cells and to 25 ± 4% of CD3^+^CD56^−^ T cells (Figure 4D). This distribution changed only slightly during IL-2 stimulation up to 66 and 33%, respectively. Moreover, the percentage of residual T cells did not increase as shown exemplarily in Figure 2B.

The viability of cryopreserved NK cells after unfreezing was significant higher for IL-2 stimulated NK cells (median 60%, range: 25–91%) compared to unstimulated NK cells (median 30%, range: 17–55%) as shown in Figure 4E. The difference was even meaningful and highly significant for the NK cell recovery after unfreezing resulting in a median of 84% (range 75–92%) for IL-2 stimulated NK cells compared to a median of 22% (range 8–44%) for unstimulated NK cells, respectively.

As opposed to the large quantities of monocytes contained at the start of stimulation (>4% in half and >10% monocytes in another 3 of 40 cell suspensions after immunomagnetic isolation, Figure 3F, the content of monocytes decreased dramatically during IL-2 stimulation attributable to adhesion to the bags. Therefore, the purity of the final suspensions increased to >98% CD56^+^CD3^−^ NK cells after stimulation.

### NK cell cytotoxicity

**Figure 5:**
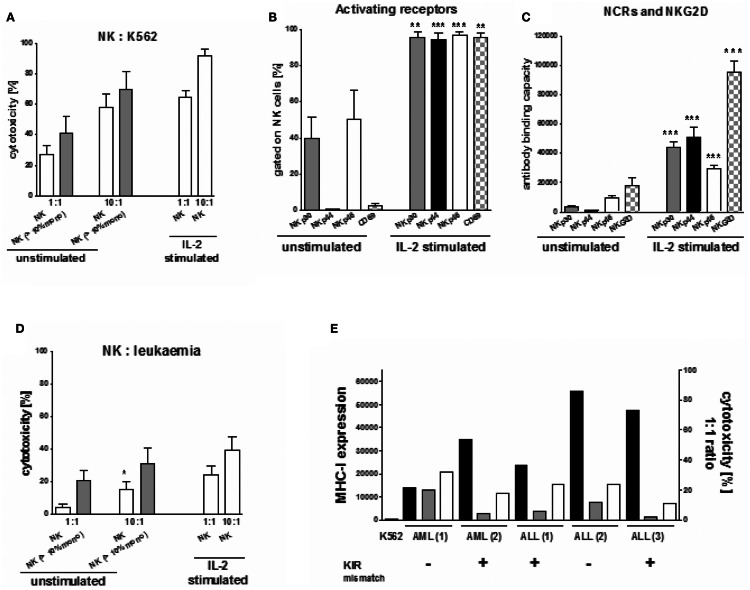
**Cytotoxicity of NK cells against K562 and patients’ leukemic cells**. **(A)** Donor NK cell cytotoxicity against K562 cells was significantly increased after IL-2 stimulation compared to unstimulated NK cells and also seemed to be slightly increased for unstimulated NK cells if the purified NK cell products include >10% concomitant monocytes. **(B)** IL-2 stimulation led to a significant increase in the percentage of NK cells with the natural cytotoxicity receptors NKp30, Nkp44, NKp46, and the activating receptor CD69. **(C)** In addition IL-2 stimulation led to a significant up-regulation of NKp30, NKp44, NKp46 on NK cells as well. **(D)** Concomitant monocytes (>10%) in the purified NK cell products increased donor NK cell cytotoxicity against the haploidentical leukemic blasts of pediatric patients with leukemia (1 AML, 1 ALL) significantly compared to those with a low amount or no monocytes (1 AML, 2 ALL). This improved cytotoxicity was compared to those of IL-2 stimulated NK cells against the respective haploidentical leukemic cells. **(E)** The leukemic blasts of five pediatric patients (3ALL, 2 AML), in which the interaction between the haploidentical donor NK cells and the respective individual patients’ leukemic cells was investigated, all showed a significantly increased high expression of MHC-I (black bars, measured as antibody binding capacity) compared to the MHC-I negative K562 cells. In those, in case of one ALL and one AML no KIR ligand mismatch between the haloidentical donor NK cells and the respective leukemic blasts could be demonstrated. Interestingly, cytotoxicity of unstimulated NK cells (gray bars) was found to be slightly higher in case of no compared to those with KIR mismatch. IL-2 stimulated NK cells showed an improved cytotoxicity (white bars).

Natural Killer cell cytotoxic activity of both the freshly isolated NK cells of all donors (*n* = 40) and of the IL-2 stimulated NK cells of 17 donors was tested against K562 cells. Additionally, when available, the NK cells of five donors were tested against the respective recipient’s leukemic cells. Unstimulated NK cells killed a median of 28% and of 58% target cells in the 1:1 and 10:1 NK:K562 ratios, respectively (Figure 5A). The lytic NK cell activity increased after IL-2 stimulation, reaching a median cytotoxicity of 64 and 92% in the two ratios. Concomitantly, the surface expression of the activating receptors CD69, NKG2D, and the NCRs NKp30, NKp44, and NKp46 was up-regulated (Figures 5B,C). Both, a significant IL-2 driven increase of the percentage of NK cells expressing NCR and of the surface density of NCR were shown. Further, the density of NKG2D increased as found by ABC *p* < 0.01 or *p* < 0.001. Interestingly, the three unstimulated NK cell suspensions with monocytes of >10% also exerted higher cytotoxic activity against K562 cells (70 and 41%) in the 10:1 and 1:1 effector:target ratio compared to those with a smaller amount of monocytes reaching a median NK cell cytotoxicity of 58 and 27%, respectively (Figure 5A). However, this was not statistically significant.

We also investigated the interaction between the haploidentical NK cells and the leukemic cells of five children (three with ALL, 2 with AML), Again, a high percentage of monocytes led to enhanced NK cell lysis of the leukemic cells (1 ALL, 1 AML) in both the 10:1 and 1:1 effector:target ratio compared to those cell suspensions containing no or only a small amount of monocytes (Figure 5D). We measured a killing of 31 and 16% in the 10:1 and 1:1 ratio, respectively, compared to 21 and 4.5% for the three NK cell suspensions (2 ALL, 1 AML) containing less than 10% monocytes. This difference was significant in the 1:1 ratio (Figure 5D; *p* < 0.05). This increased cytotoxicity seemed to be independent of both the MHC-I expression of the leukemic cells and the KIR mismatch in GvL direction. Interestingly, in both cases with a higher killing activity of unstimulated NK cells, no KIR mismatch was demonstrated between donor and recipient as opposed to the three pairs with lower killing that displayed one or two KIR ligand mismatches (Figure 5E). IL-2 stimulated NK cells led to an improved killing activity against patients’ leukemic cells for both donor:recipient pairs with and without KIR mismatch.

In these, both patients with ALL lacked one HLA-C ligand for donor group I KIRs, while the AML patient had a missing HLA-C donor KIR group II ligand and a missing HLA-A11. Moreover, in all 5 investigated samples, the leukemic cells showed a high MHC-I expression compared to K562 cells (Figure 5E), suggesting that they would be resistant against NK cell lysis. Nevertheless cytotoxicity of the haploidentical donor NK cells with >10% concomitant monocytes against the ALL (2) sample was moderately high, although the leukemic blasts reached the highest MHC-I expression and a KIR mismatch was lacking.

## Discussion

Clinical-scale collection, enrichment, activation, and expansion of purified NK cells is feasible. However these procedures are time-consuming and expensive, need particular skills, and must be performed according to a GMP-compliant protocol. To date, NK cell trials and ongoing clinical phase I/II studies have shown the feasibility of using freshly purified or IL-2-activated donor NK cells for the treatment of high-risk patients suffering from leukemia or tumors in both non-transplant settings and after haploSCT as an additional immunotherapy (Koehl et al., 2004; Passweg et al., 2004; Miller et al., 2005; Rizzieri et al., 2010; Rubnitz et al., 2010; Curti et al., 2011; Nguyen et al., 2011; Stern et al., 2013). NK cell doses ranged from 1 × 10^6^/kg to 1 × 10^8^ CD56^+^CD3^−^ NK cells/kg BW, very often with less than 5 × 10^4^ CD3^+^ T cells/kg BW (Passweg et al., 2006; Huenecke et al., 2010; Rizzieri et al., 2010; Rubnitz et al., 2010; Brehm et al., 2011; Stern et al., 2013). These first immunotherapy trials showed that NK cells can be administered without immediate adverse events, that they were well-tolerated by the patients and did not induce severe GvHD. However, some cases of GvHD >grade II have been observed after NK cell infusion, which seemed to be associated with a less efficient T-cell depletion. Whether GvHD is attributable to contamination by T cells or is due to the effects of NK cells cannot be determined based on these clinical data so far. But the fact that – at least in some cases of GvHD – the T-cell content was higher than in cases without GvHD, seems to favor a T-cell effect.

Thus, advances in NK cell therapy after haploSCT requires both, refined manufacturing procedures to obtain NK cells products with a minimum of residual T cells and the development of dependable methods to obtain adequate numbers of effector cells. In the present study we report on 40 GMP-conform NK cell products manufactured by using immunomagnetic CD3 T-cell depletion, followed by a CD56 cell enrichment step with modifications in the T-cell removal. The median T-cell depletion was significantly better using a single or double procedure of the time-consuming depletion program depletion D2.1 compared to the fast depletion program D3.1, but median recovery of CD56^+^CD3^−^ NK cells was inversely correlated. A number of various studies have shown that clinical-scale NK cell isolation from non-stimulated leukapheresis products, using CD3^+^ cell depletion/CD56^+^ cell enrichment, lead to highly purified CD56^+^CD3^−^ NK cell products with a median purity ranging from 90 to 98.6% (Iyengar et al., 2003; Lang et al., 2003; Passweg et al., 2004; Koehl et al., 2005; McKenna et al., 2007; Meyer-Monard et al., 2009; Rizzieri et al., 2010). The high NK cell purity and extensive T-cell depletion was possible at the expense of considerable loss of NK cells during isolation. The final recovery of CD56^+^CD3^−^ NK cells ranged between 20 and 58% in these different studies and we could demonstrate improved recovery of NK cells to a median of 68% using the fast depletion program 3.1. Overnight storage of the leukapheresis product resulted in a greater loss of NK cells during the NK cell selection process compared to processing of fresh harvests as we could show previously (Meyer-Monard et al., 2009). For over night storage cells have been placed in the GMP facility on a waver at 4°C in the dark. A much higher NK cell recovery was also obtained by using only a CD3^+^ cell depletion step, without further CD56^+^ cell enrichment. However, such a product was associated with low purity and less T-cell depletion (McKenna et al., 2007, 2012; Koepsell et al., 2013). Similarly, the final T-cell number was much higher if a CD56^+^ cell selection was used alone (Rizzieri et al., 2010). In contrast, the two-step NK cell product manufacturing described here led to efficient T-cell depletion for haploSCT, and this could be further increased by performing the CD3^+^ cell depletion step twice. A residual T-cell contamination between below the detection limit (0.001%/0.1/μl) and 0.09% in the final product allows the infusion of NK cell products of more than 1.0 × 10^7^ CD56^+^CD3^−^ NK cells/kg BW with less than 5.0 × 10^4^ CD3^+^ cells/kg BW, and often even less than 2.5 × 10^4^ CD3^+^ T cells/kg BW (Brehm et al., 2011; Stern et al., 2013). Therefore, for future NK cell purification, we recommend a combination of the fast depletion program 3.1 in order to deplete most of the T cells and if necessary followed by a second round or T-cell depletion using the effective depletion program 2.1 which is not time-consuming for a starting percentage of 0.5% T cells.

The objective of NK cell purification is not only to remove potentially unwanted T cells, but also to enable activation and expansion of the NK cells. Indeed, enriched NK cells can be infused without any additional manipulation, or after overnight culture with a high-dose of IL-2. They can also be expanded with IL-2 or other cytokines, such as IL-15, alone or in combination for two to several weeks in cell culture bags or in a bioreactor (Koehl et al., 2005; Sutlu et al., 2010). Similarly, it is possible to expand single KIR^+^ NK cells (Siegler et al., 2010). To date, there is evidence that a combination of multiple cytokines, such as IL-2, IL-12, IL-15, Il-18, and IL-21, may further increase cytotoxic activity of NK cells. Other protocols reach a very high NK cell expansion rate using genetically modified K562 cells (Shook and Campana, 2011; Lapteva et al., 2012). In addition to NK cell enrichment from leukapheresis products, NK cells can also be generated from cord blood (Spanholtz et al., 2010). However, there are several limitations in regard to GMP-conform protocols such the lack of marketing authorization for the respective clinical-scale cytokines or the use of gene-manipulated tumor cells.

Therefore, clinical-scale *in vitro* expansion has two aims, to activate the selected CD56^+^CD3^−^ cells, and to increase the total number of NK cells. In the present study we present our clinical-scale protocol that enables the generation of NK cells in a closed bag system conforming to GMP guidelines. We could show that highly enriched CD56^+^CD3^−^ NK cells could be expanded under the influence of 1000 U/ml IL-2, although we observed a lag of 3–4 days before the remaining NK cells started to proliferate. Between day 4 and 6, expansion occurred, but with marked differences in the proliferation rate among 17 NK cell donors. While NK cells of two donors reached the starting NK cell level only, in 12 cases a median fourfold and in two cases a 30-fold increase of CD56^+^CD3^−^ NK cells could be observed after 12 days. This reflects the strong differences with regard to biological material of various donors. Although NK cells were viable immediately after purification (median 94%), the vital NK cell count decreased by 30–65% during the first 3–4 days following IL-2 stimulation, but viability recovered completely toward the end of the expansion period. Purity of the CD56^+^CD3^−^ NK cells was higher at the end of the expansion period (>98%) compared to the first analysis performed directly after purification. Interestingly, this effect was due to the amount of concomitant monocytes (or other antigen-presenting cells like DCs) in the NK cell products after purification, but it was lost during cell expansion. Importantly, no overgrowth of the remaining T cells was observed during expansion and activation with IL-2. The phenotype of the residual T cells did not change significantly during expansion regarding to CD56^+^CD3^+^ NK-like T cells and CD3^+^CD56^−^ T cells. In contrast, we found large differences between CD16^+^ and CD16^dim/−^ NK cell subpopulations at the end of the expansion period compared to a homogeneous distribution between immunoregulatory and cytotoxic NK cells after purification as described previously more in detail (Huenecke et al., 2010).

As cytotoxic quality control, in our present study unstimulated NK showed a sufficient lytic activity against the MHC-I^negative^ cell line K562, that significantly increased under the influence of IL-2. The demonstrated up-regulation of the NCRs NKp30, NK44, NKp46, the activating receptor CD69 as well as of NKG2D, and the increase in intracellular pSTAT3 and AKT signaling might explain the improved NK cell cytotoxicity when cultured in IL-2. Interestingly, this strongly increased NCR and NKG2D expression overrode important inhibitory mechanisms which allowed IL-2 activated haploidentical NK cells to lyse MHC-I^positive^ targets as we demonstrated for the respective leukemic blasts of five pediatric patients suffering from AML and ALL. In contrast, we expected no or a very weak cytotoxicity of unstimulated NK cells against MHC-I^positive^ leukemia. Surprisingly, in two cases a significantly higher NK cell cytotoxicity of unstimulated NK cells against these respective MHC-I^positive^ leukemia’s was found if the cell count of concomitant monocytes was above 10% of the cell product. Most importantly though, this improved lysis occurred in donor:recipient pairs without KIR mismatch in GvL direction. Similarly, we had demonstrated previously an increased cytotoxicity of haploidentical unstimulated NK cells with concomitant DCs and monocytes against neuroblastoma compared to IL-2 stimulated NK cells (Kloess et al., 2010). Our observations indicate that in addition to the well-known balance between inhibitory and activating receptors to lyse MHC-I^negative^ targets by unstimulated NK cells, other mechanism might override the influence of inhibitory receptors such as KIRs leading to (i) changed balance for unstimulated NK cells under the influence of antigen-presenting cells and (ii) enhanced effects of activating signals only if activated NK cells cause also lysis of MHC-I^positive^ targets. However our given examples are very few and need further investigations. Nevertheless, in line with our results are observations of Obeidy and Sharland (2009) and Vivier et al. (2012) reviewing the importance of both up-regulation of NKG2D and the NK cell environment which can lead to killing of MHC-I^positive^ targets by NK cells.

Future studies should improve NK cell immunotherapy by increasing the understanding of the conditions leading to tumor cell killing by NK cells, by increasing the cytotoxicity of NK cells against various malignancies, and by optimizing the schedule of the NK administration based on results of ongoing phase I/II studies. Given the plausible benefit of IL-2-stimulated NK cells compared to freshly isolated, unstimulated NK cells with regard to cytotoxicity, it may be possible to increase the killing capacity of NK cells by improving the cross-talk with antigen-presenting cells like DCs or simply monocytes. DCs and NK cells specialize in complementary functions, including IL-12 or IFN-α/β secretion and antigen presentation for the former, and IFN-γ secretion and killing of infected or tumor cells for the latter. Thus, the outcome of NK-DC crosstalk is likely to increase lytic activity against malignancies compared to NK cell cytotoxicity alone (Walzer et al., 2005; Pallandre et al., 2008; Wehner et al., 2009; Jacobs and Ullrich, 2012). Additional investigations are necessary to develop strategies to overcome tumor immune escape mechanisms. In pediatric patients with neuroblastoma tumor cells escaped from immune surveillance by releasing of soluble MICA (ligand MHC class I-chain-related gene A) compromising NKG2D-dependent NK cell cytotoxicity. Elevated sMICA levels in those patients’ plasma correlated significantly with impaired NK-cell mediated cytotoxicity of the infused haploidentical donor NK cells (Kloess et al., 2010). This could be overcome in part by higher numbers of NK cells which stress the importance of developing improved NK cell purification and expansion techniques, especially if multiple NK cell applications are required. Options may encompass development of MAb against sMICA or genetic engineering of NK cells by introduction of chimeric receptors for tumor retargeting (Esser et al., 2012). Other strategies focus on enhancing tumor cell recognition by using small interfering RNA to silence inhibitory receptors or by expansion of tumor-reactive NK cells.

In order to improve NK cell purification we recommend a combination of the fast depletion 3.1 program followed by a second run of T-cell depletion using the slow, but effective depletion 2.1 program for removing of residual T cells securely. After the final CD56 enrichment step for the following GMP-conform expansion of CD56^+^CD3^−^ NK cells, IL-2 activation might be improved by the additional use of antigen-presenting cells such as monocytes or DCs.

## Conflict of Interest Statement

The authors declare that the research was conducted in the absence of any commercial or financial relationships that could be construed as a potential conflict of interest.

## Author Note

Conceived and designed the experiments: Ulrike Koehl, Claudia Brehm, Sabine Huenecke, Stefanie-Yvonne Zimmermann, Stephan Kloess, Ruth Esser. Performed the experiments: Claudia Brehm, Sabine Huenecke, Stefanie-Yvonne Zimmermann, Melanie Bremm, Tanja Gardlowski, Andrea Quaiser, Stephanie Erben, Claudia Wunram, Eileen Auth, Ulrike Koehl, Ruth Esser. Contributed materials and analysis: Torsten Tonn, Christian Seidl, Ulrike Koehl, Ruth Esser. Wrote the paper: Ulrike Koehl. Revised the manuscript: Claudia Brehm, Sabine Huenecke, Stephan Kloess, Melanie Bremm, Evelyn Ullrich, Eileen Auth, Sandrine Meyer-Monard, Martin Stern, Ruth Esser. Provided clinical data: Jakob Passweg, Jan Soerensen, Martin Stern, Sandrine Meyer-Monard, Thomas Klingebiel, Peter Bader, Dirk Schwabe.
